# Novel pH‐Responsive Structural Rearrangement of Myristic Acid‐Conjugated Quetiapine Nanosuspension for Enhanced Long‐Acting Delivery Performance

**DOI:** 10.1002/advs.202405200

**Published:** 2024-09-03

**Authors:** Hy Dinh Nguyen, Hai Van Ngo, Beom‐Jin Lee

**Affiliations:** ^1^ Department of Pharmacy, College of Pharmacy Ajou University Suwon 16499 Republic of Korea; ^2^ Institute of Pharmaceutical Science and Technology Ajou University Suwon 16499 Republic of Korea

**Keywords:** initial burst release, long‐acting injectables, nanoaggregates, pH‐responsive structural rearrangement, quetiapine myristate, self‐assembled nanoparticles

## Abstract

Quetiapine myristate (QM), an ester‐bonded lipophilic prodrug of quetiapine (QTP), is synthesized and converted into an amphiphilic structure in acidic pH to trigger a novel self‐assembled QM nanosuspension (QMN). Following injection, this QMN rearranges within physiological pH to form nanoaggregates in structure, resulting in enhanced physicochemical properties and in vivo therapeutic performance without an initial burst release. The 200‐nm‐sized QMN exhibits less invasive injection, higher drug content, and better storage stability profile than conventional poly(lactide‐co‐glycolide) (PLGA) nanosuspensions containing QTP or QM. Following a single intramuscular injection to beagle dogs (35 mg kg^−1^ QTP), QMN undergoes pH‐responsive nanoaggregation to form the lipophilic prodrug, providing esterase‐oriented sustained release for five weeks compared with the two‐week period of PLGA nanosuspensions. Notably, QMN exhibits improved in vivo pharmacokinetic performance with long‐acting delivery while minimizing issues associated with polymeric PLGA formulations, including the initial massive burst release, cellular toxicity, and adverse side effects. These results support the further development of QMN as a novel long‐acting injectable to improve patient compliance and dosing frequency.

## Graphical abstract

This study investigates in vitro and in vivo performance of a pH‐responsive nanosuspension of quetiapine myristate (QMN) as a novel long‐acting injectable. QMN offers simple formulation design and innovative release mechanism that can address challenges associated with polymeric systems, providing high drug content, high stability, low toxicity, and long‐acting performance without large initial burst release.

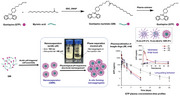

## 1 Introduction

Long‐acting injectables (LAIs) have proven advantageous over oral dosage forms with superior patient compliance and treatment efficacy.^[^ ^1^, ^2^ ^]^ However, challenges remain in LAI development, including overcoming trypanophobia (extreme fear of needles), improving the low drug loading capacity, consistency of in vivo performance, and safety issues regarding the initial massive burst release, and simplifying the manufacturing process.^[^ ^2^, ^3^ ^]^ Many polymeric LAIs have a large initial burst release that often consumes a quarter of the total drug on the first day with severe adverse effects.^[^ ^4^ ^]^ Additionally, using large‐diameter gauge needles to accommodate large particle diameters can cause trypanophobia and reduce patient acceptance. For example, the size of poly(lactide‐co‐glycolide) (PLGA) microspheres loaded with risperidone (Risperdal Consta) ranges from 25 to 180 µm, requiring the use of a 21‐gauge needle with an outer diameter of 0.813 mm. Therefore, LAI formulations that can be administered using thinner needles will undoubtedly make clinical use more patient‐friendly.^[^ ^4^ ^]^ Specifically, this can be achieved via nanosuspensions (NSPs) that deliver considerable amounts of active drugs without significantly affecting the system's viscosity, allowing for a thinner gauge needle.^[^ ^5^, ^6^ ^]^ However, the current preparation methods for NSPs require complicated pharmaceutical techniques for particle size reduction (wet milling or high‐pressure homogenization) with the potential risk of erosion of the milling material.^[^ ^7^, ^8^ ^]^ In addition, using stabilizers or surfactants to improve thermodynamic stability can cause tissue irritation and other adverse side effects.^[^ ^2^ ^]^

Quetiapine (QTP) is a first‐line antipsychotic drug for the treatment of schizophrenia; however, lifelong daily administration of QTP tablets is associated with a low patient acceptance profile. Accordingly, substituting oral dosage forms with LAIs can potentially improve patient compliance and treatment effectiveness. However, research on LAIs of QTP has rarely been reported. PLGA microspheres loaded with norquetiapine—an active metabolite of QTP—for LAI formulations had relatively larger particle size (8.86 µm), low drug content (<10%) and low loading efficiency (<50%). Following intramuscular (IM) injection into rats, the formulation achieved ten days of drug release, with a massive burst effect after the first day and a rapidly decreasing plasma concentration below the minimum effective concentration (MEC) within a few days.^[^ ^9^ ^]^ Hence, to achieve clinically accessible LAI of QTP, the formulation must require a smaller needle gauge, minimize the burst release, and maintain plasma concentrations over an extended period.

Our research group utilizes a fattigation platform to chemically conjugate biomacromolecules, such as albumin, gelatin, and peptide drugs with fatty acids.^[^ ^10^, ^11^ ^]^ The obtained amphiphilic structures self‐assemble in aqueous solutions, serving as drug nanocarriers or self‐assembled prodrugs to improve drug physicochemical properties and biopharmaceutical performance.^[^ ^12^, ^13^ ^]^ This strategy can be applied in LAIs to increase the lipophilicity of target molecules, forming a lipophilic prodrug that can be formulated as NSPs for extended‐release profiles.^[^ ^8^, ^14^ ^]^ In our previous study, a lipophilic QTP–myristic acid conjugate or quetiapine myristate (QM) was synthesized, and the 200‐nm self‐assembled QM NSP (QMN) obtained via pH‐triggered self‐assembly of QM in a pH 1.2 buffer represents a potential LAI formulation.^[^ ^15^ ^]^ This nanonization platform is based on the pH‐dependent solubility of QTP and QM, offering a simple formulation without surfactants and a straightforward preparation process without high‐shear grinding machine usage.

**Scheme 1:**
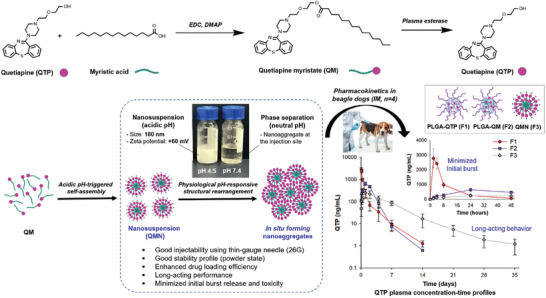
Schematic illustration of pH‐responsive myristic acid‐conjugated quetiapine nanosuspension (QMN) for enhanced in vitro and in vivo performance and long‐acting delivery. Quetiapine myristate (QM), a lipophilic prodrug synthesized from quetiapine (QTP) and myristic acid, can be converted to QTP by plasma esterase. In an acidic buffer, QM self‐assembles to form positively charged 200‐nm sized QMN with good injectability, stability, and drug loading efficiency. Following intramuscular injection in beagle dogs, QMN undergoes physiological pH‐responsive structural rearrangement, forming nanoaggregates at the injection site for long‐acting performance with minimized initial burst release compared with conventional poly(lactide‐co‐glycolide) (PLGA) nanosuspensions loaded with QTP or QM.

In this study, we compared the physicochemical properties and in vivo performance of QMN with conventional polymeric formulations (PLGA NSPs loaded with QTP or QM). As illustrated in **Scheme** **1**, QMN exhibited enhanced physicochemical and biopharmaceutical properties, offering high drug‐loading efficiency, good injectability and stability, local tolerability, and minimized initial burst effect. QMN underwent physiological pH‐responsive structural rearrangement to form nanoaggregates at the injection site, providing a five‐week sustained release profile. This platform has the potential to be developed for human use to improve frequent dosing and patient compliance. Collectively, our work provides proof‐of‐concept for pH‐responsive NSPs as a novel LAI with advanced in vitro and in vivo performance. This can be adapted for various drugs with pH‐dependent solubility, such as haloperidol, fluphenazine, zuclopenthixol, paliperidone, nalmefene, and nalbuphine, to improve treatment of chronic diseases and prevalent health issues.

## 2 Results and Discussion

### 2.1 Preparation and Characterization of NSPs

#### 2.1.1 Particle Size, Zeta Potential and Morphology

**Figure 1:**
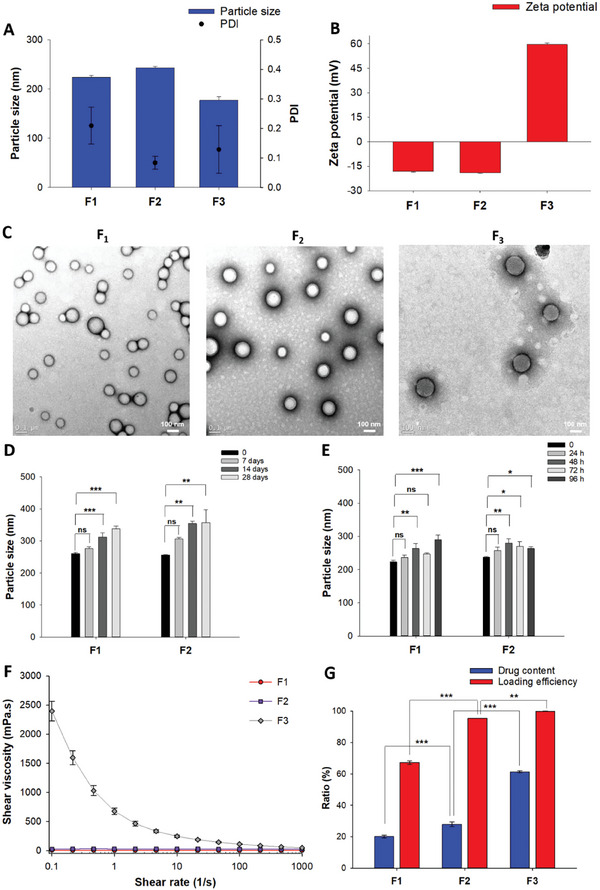
Physicochemical properties of three nanosuspensions (F1–F3). A) Particle size, polydispersity index (PDI) and B) zeta potential of nanosuspensions. C) Representative TEM images of nanosuspensions. D,E) Physical stability of polymeric nanosuspensions (F1, F2) in the powder (D) and aqueous liquid (E) states at 25 °C. F) Shear viscosities of nanosuspensions as a function of shear rate. G) Drug content and loading efficiency of three nanosuspensions. Data are presented as mean ± standard deviation (*n* = 3). Significant differences were analyzed using one‐way analysis of variance (ANOVA) followed by the Bonferroni post hoc test: ***p < 0.001, **p < 0.01, and *p < 0.05; ns: no significant difference (*p* > 0.05).

Two polymeric formulations, including PLGA NSPs loaded with QTP (PLGA‐QTP NSPs, F1) and QM (PLGA‐QM NSPs, F2), were prepared to compare with QMN (F3). The three NSPs were fabricated with similar particle sizes (≈150–250 nm) but variable surface properties (**Figure** **1**A,B). PLGA NSPs (F1 and F2) had negatively charged surfaces with zeta potential values of ≈ –20 mV, agreeing with previous publications.^[^ ^16^ ^]^ On the other hand, QMN (F3) bore a positive charge of +59.57 mV, as the nanoparticles were obtained from the self‐assembly of protonated QM molecules in an acidic buffer solution. The self‐assembly of QMN occurs at QM concentrations above the critical micelle concentration (CMC) of 0.1175 mg mL^−1^.^[^ ^15^ ^]^ Figure 1C shows the morphological images of the NSPs measured by field emission–transmission electron microscopy (FE‐TEM). The TEM images created by the transmitted electrons passing through the NSPs showed that the nanoparticles were evenly distributed with a spherical shape.

#### 2.1.2 Physical Stability of Three NSPs

The stability of the three formulations was evaluated in the aqueous liquid state (injectable form) and freeze‐dried powder (storage form). We previously reported the high stability of QMN (F3), with no significant change in particle size (≈180 nm) for the lyophilized powder over a four‐week storage period and aqueous nanosuspension over 96 h at 25 °C.^[^ ^15^ ^]^ In contrast, the particle size of F1 and F2 slightly increased from ≈250 to 350 nm over four weeks of lyophilized powder storage at 25 °C, indicating the gradual aggregation of PLGA nanoparticles. A similar trend was observed after reconstitution in the liquid‐injectable form after 96 h at room temperature (Figure 1D,E).

The variations in surface properties might account for the differing stability among the formulations in which the electrostatic stabilizing effect was stronger than the steric effect.^[^ ^17^ ^]^ That is, while PLGA NSPs (F1 and F2) were stabilized by the steric mechanism with the poly(vinyl alcohol) (PVA) surface coating, positively charged QMN (F3) retained a nano‐size primarily due to electrostatic repulsion. The aggregation of polymeric nanoparticles could lead to the formation of a larger cluster over long‐term storage, potentially causing blockages in the needle and impacting injectability. Conversely, the good stability profiles of QMN in solid and liquid states highlighted its stable physicochemical properties during storage and the reconstitution process prior to injection.

#### 2.1.3 Viscosity and Injectability

Viscosity is an essential parameter in drug formulation, impacting administration, stability, and drug release kinetics. A high viscosity creates significant challenges in injectability as it requires high injection force and large needle size, causing patient discomfort.^[^ ^18^ ^]^ At a low shear rate of 0.1 s^−1^, the viscosity of PLGA NSPs was ≈10 mPas (F1) and 30 mPas (F2), while QMN possessed a high viscosity of ≈2500 mPas. The relatively high viscosity of QMN at static conditions alleviated the mobility and aggregation of nanoparticles, which might contribute to the better stability profile of QMN relative to polymeric NSPs. While the viscosity of F1 and F2 remained relatively unchanged upon an increased shear rate, the viscosity of F3 gradually decreased to ≈45 mPas at a 10 000 s^−1^ shear rate, resembling the shear rate inside the needle upon application of the injection force (Figure 1F).

This non‐Newtonian shear‐thinning behavior of QMN is due to the micelle‐like structure of the nanoparticles, similar to other self‐assembled surfactant structures.^[^ ^19^ ^]^ Amphiphilic molecules generally self‐assemble to form globular micelles at low concentrations. However, upon an increase in QMN concentration to >100 mg mL^−1^ (**Table** **1**), the nanoparticles might overlap and become entangled, rearranging to form a three‐dimensional dense network due to frictional interactions and mechanical interference between amphiphilic molecules via intermolecular forces, such as hydrogen bonding and electrostatic interactions.^[^ ^19^ ^]^ Meanwhile, when QMN is subjected to the injection force through a needle, the three‐dimensional network is temporarily dismantled due to the shear‐induced alignment of nanoparticles along the flow direction and the disruption of intermolecular interactions, leading to reduced viscosity and improved injectability.^[^ ^20^, ^21^ ^]^ Interestingly, the viscosity of QMN was recovered upon stress release (Figure S1, Supporting Information) with the recovery of the gel‐like network. This might improve drug retention at the injection site, contributing to the long‐acting performance of QMN and minimizing the burst release effect.^[^ ^22^ ^]^

**Table 1:** Concentration and physical behaviors of three nanosuspensions (F1–F3) in pH 4.5 and pH 7.4 buffers.

Formulation	F1 (PLGA‐QTP)		F2 (PLGA‐QM)		F3 (QMN)	
**Concentration of QTP/QM** [mg mL^−1^]	69.6 ± 1.2		109.5 ± 0.8^a^ ^)^		101.5 ± 1.4^b^ ^)^	
**pH buffer**	pH 4.5	pH 7.4	pH 4.5	pH 7.4	pH 4.5	pH 7.4
**Physical state**	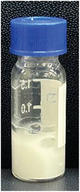	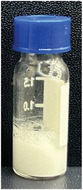	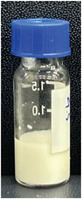	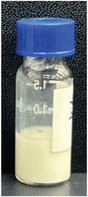	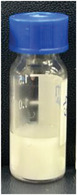	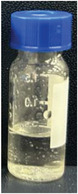
	Suspended	Suspended	Suspended	Suspended	Suspended	**Nano‐aggregated**

^a)^ QM concentration in F2 equivalent to 70.7 ± 0.5 mg mL^−1^ QTP

^b)^ QM concentration in F3 equivalent to 65.6 ± 0.9 mg mL^−1^ QTP. Data are expressed as mean ± standard deviation (*n* = 3).

The injectability of the three NSPs (F1–F3) was evaluated using a thin‐gauge (26G) needle, which is considerably narrower than the 21G needle used clinically for PLGA microsphere products. All NSPs passed readily through the needle without requiring high pressure (Video S1, Supporting Information). Hence, these formulations can be administered with minimal discomfort to the patient.

#### 2.1.4 Drug Content and Loading Efficiency

By using the bottom‐up approach, i.e., the self‐assembly of QM in an acidic buffer solution, no drug loss occurred during F3 (QMN) preparation. In contrast, the preparation of PLGA nanoparticles as drug carriers resulted in lower loading efficiency for F1 (67.18%) and F2 (95.36%) (Figure 1G). At different equal drug ratios initially mixed with PLGA, QM was encapsulated into the PLGA NSPs more effectively than QTP (Table S1, Supporting Information) due to its higher lipophilicity.

Regarding the drug content, F1 and F2 had relatively low ratios (<30%) due to the large amounts of polymers (PLGA and PVA) in the formulations. In contrast, the main composition of QMN was QM, accounting for ≈95% of the solid weight, equivalent to 61.31% QTP (the remaining 5% was HCl as a pH modifier). Therefore, the drug content of F3 was two and three times higher than those of F2 (27.92%) and F1 (20.12%), respectively. A higher drug content requires fewer nanoparticles and a smaller injection volume to reach the desired dose.^[^ ^4^ ^]^ Overall, the lipophilic drug–fatty acid conjugate offers a higher loading efficiency into NSPs than the original drug, and the pH‐triggered self‐assembly can improve the drug content and loading efficiency in the final formulation.

### 2.2 pH‐Responsive Property of QMN

A parenteral aqueous solution should ideally have a pH close to physiological pH (7.35–7.45) to avoid pain, phlebitis, and tissue necrosis. However, as a reasonably wide pH range can be tolerated by diluting with the blood at the injection site, the pH of most licensed parenteral solutions is maintained between 3 and 9 to meet other requirements, such as drug solubility and product stability.^[^ ^23^, ^24^ ^]^ All three NSPs had slightly acidic pH values within an acceptable range for IM formulation (F1: 5.21 ± 0.13, F2: 5.09 ± 0.07, and F3: 3.97 ± 0.15), minimizing the local pain caused by the unphysiological pH.^[^ ^25^ ^]^ The physical state of the three NSPs was investigated in pH 4.5 and 7.4 buffers, representing the state of nanosuspensions before and after IM injection, respectively. As expected, the physical state of F1 and F2 remained unchanged in these buffers. In contrast, F3 exhibited nanoparticle aggregation in pH 7.4 and retained a dispersed state in the pH 4.5 buffer (Table 1).

**Figure 2:**
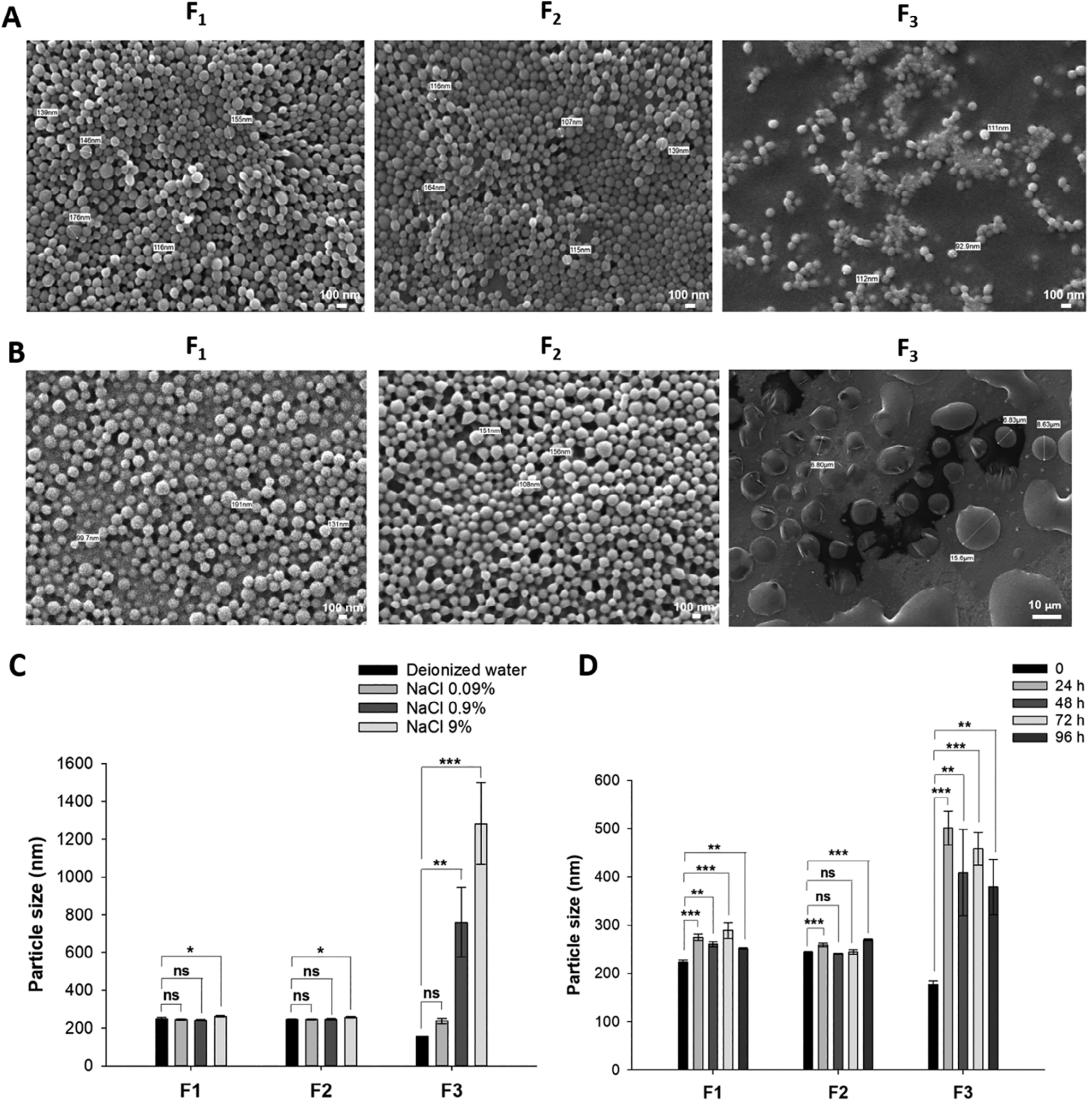
Physiological responsiveness of three nanosuspensions. A, B) SEM images of nanosuspensions (F1–F3) in pH 4.5 (A) and pH 7.4 (B) buffers. C, D) Stability of three nanosuspensions in different concentrations (w/v) of sodium chloride (NaCl) solutions (C) and fetal bovine serum (10% v/v in PBS 0.01 M) (D) at 37 °C. Data are presented as mean ± standard deviation (*n* = 3). Significant differences were analyzed using one‐way analysis of variance (ANOVA), followed by the Bonferroni post hoc test: ***p < 0.001, **p < 0.01, and *p < 0.05; ns: no significant difference (*p* > 0.05).

The nanoparticle morphologies in the two pH buffers were measured using the field emission–scanning electron microscopy (FE‐SEM) method to confirm the pH‐responsiveness of QMN. At pH 4.5 and 7.4, PLGA nanoparticles remained globular with smooth surfaces ranging from 100 to 200 nm. In contrast, when the pH changed from 4.5 to 7.4, QMN experienced pH‐responsive precipitation to form nanoaggregates, with particles increasing from 100–200 nm to 5–10 µm (**Figure** **2**A,B). The formation and particle size distribution of QMN were pH‐dependent due to the protonation of QM at acidic pH.^[^ ^15^ ^]^ Therefore, an environment with a pH above the pKa of QTP (3.3 and 6.8) can precipitate QMN as nanoparticles become uncharged and aggregated.

When nanoparticles are in contact with biological fluids, the complex environment regulated by pH, salt, and proteins can cause nano‐aggregation.^[^ ^26^, ^27^ ^]^ Therefore, the stability of the three nanosuspensions in salt and serum conditions, mimicking physiological conditions, was also evaluated to predict their state within the body after injection. Sodium chloride (NaCl) solutions (0.09%, 0.9%, and 9%, w/v) were selected to investigate the effect of electrolytes on the colloidal stability of the three nanosuspensions. PLGA NSPs exhibited nearly unchanged particle sizes in different salt concentrations. In contrast, QMN became larger as a function of salt concentrations due to the reduced or loss of electrostatic repulsion between nanoparticles,^[^ ^27^ ^]^ particularly at concentrations ≥0.9% (physiological level; Figure 2C).

The nanosuspension stabilities in serum were assessed in 10% fetal bovine serum (FBS) diluted in phosphate‐buffered saline (PBS, 0.01 M, pH 7.4). In serum, all formulations (F1–F3) exhibited increased nanoparticle diameters due to the adsorption of serum proteins and surface charge neutralization in neutral pH. The particle size change in serum was more significant for F3 than F1 or F2 (Figure 2D). The steric hindrance of PVA on negative‐charged PLGA nanoparticles could mitigate the protein corona formation. Meanwhile, positive‐charged nanoparticles like QMN attract the negatively charged serum proteins, such as albumin, resulting in nano‐aggregation.^[^ ^27^ ^]^ The structural changes observed in the F3 formulation after injection might be advantageous for LAI as the formation of nanoaggregates at the injection site can offer a sustained drug release profile by reducing the nanoparticle surface area.

### 2.3 In Vitro Drug Release

**Figure 3:**
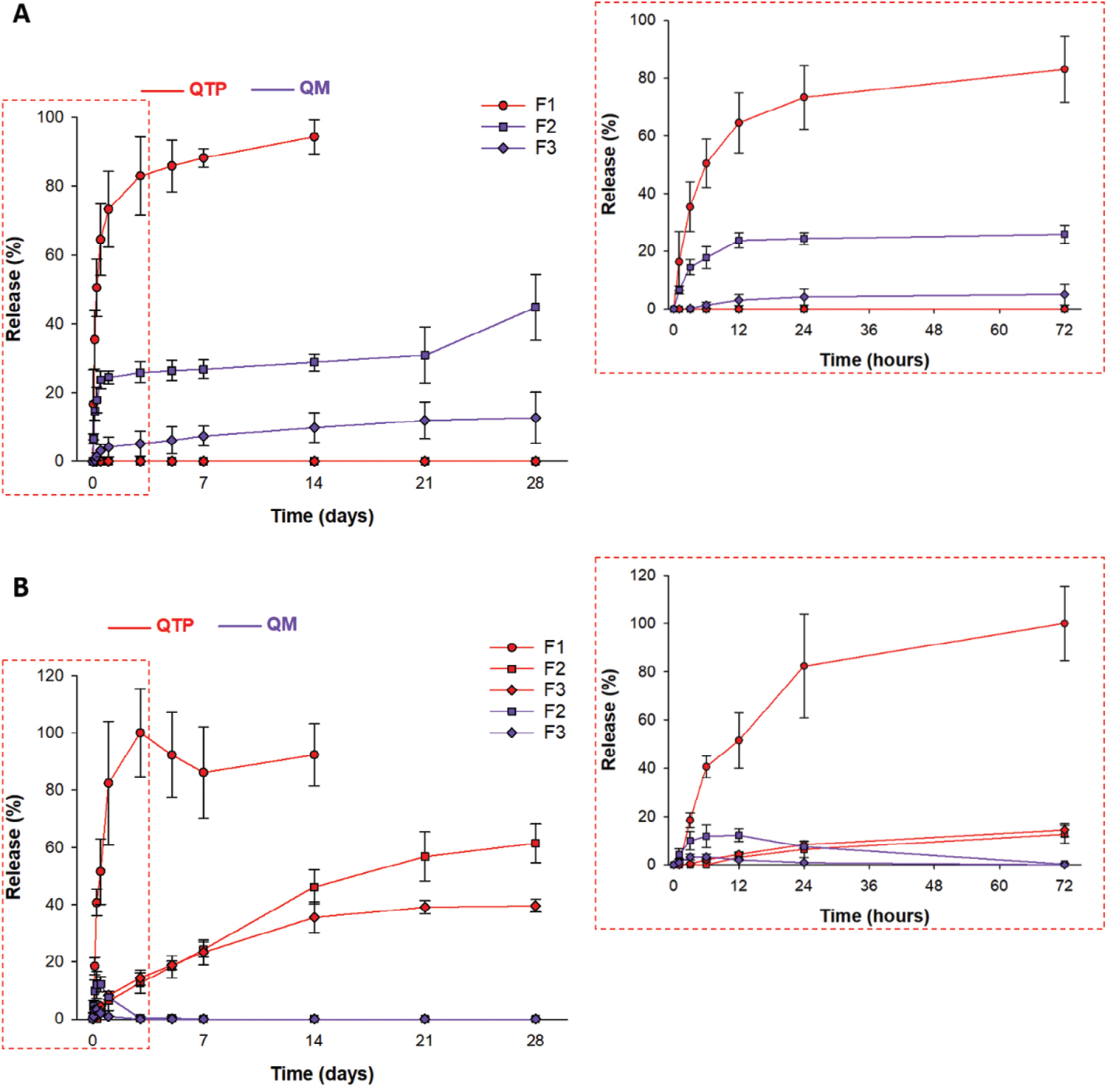
In vitro drug release profiles of NSPs (F1–F3) in the presence or absence of esterase. A) Release rate (%)–time profile of QTP (red) and QM (blue) in the absence of esterase. B) Release rate (%)–time profile of QTP (red) and QM (blue) in the presence of esterase (5 U mL^−1^). The sink condition was guaranteed by adding polysorbate 80 (0.5% w/v) to 0.01 M phosphate‐buffered saline (PBS). Data are expressed as mean ± standard deviation (*n* = 3).

The drug release test was performed in the absence and presence of esterase in the dissolution media. In esterase‐free media, F1 demonstrated a massive burst release, with over 80% of QTP released after one day. Contrarily, the lipophilic QM in F2 exhibited strong hydrophobic–hydrophobic interactions with the PLGA matrix, resulting in a controlled drug release over one month (**Figure** **3**A). The drug was released from F2 over three phases: an initial release after 12 h (≈25%), followed by a lag phase (diffusional phase) with slow drug release over 21 days (about 30%), and a secondary continuous release phase (≈45% after 28 days). This three‐phase drug release profile of PLGA‐QM NSPs was similar to that of PLGA microspheres.^[^ ^28^, ^29^ ^]^ Formulation F3 also showed slow drug release, with less than 20% of QM released after one month. The pH‐responsive structural rearrangement of QMN to form nanoaggregates at physiological pH might account for the slow drug release kinetics of F3. That is, QMN was precipitated in dissolution media (pH 7.4 buffer) to form lipophilic QM in the form of nanoaggregates with poor solubility and a slow release rate. For F2 and F3, no QTP was detected in the dissolution media as the conversion from QM to QTP required the presence of esterase.^[^ ^15^ ^]^

Esterase (5 U mL^−1^) was added to the dissolution media to mimic the enzymes present in the muscle or bloodstream.^[^ ^30^, ^31^ ^]^ In esterase‐supplemented media, the QTP concentration‐versus‐time profile was comparable for the three NSPs. Similar to the test without esterase, F1 demonstrated a faster release rate of QTP (over 80% in 24 h) than F2 or F3 (40–60% over one month). For QM‐loaded formulations (F2 and F3), the drug release was faster in the presence of esterase; the QM concentration increased on the first day and then reduced to below quantification level (0.5 µg mL^−1^) after 3 days due to the conversion of QM to QTP by esterase (Figure 3B). The QTP release rates of F2 and F3 were similar throughout the first week, although the QM concentration was higher for F2. This might be due to the hydrolysis rate from QM to QTP being slower than the release rate of QM, demonstrating the importance of the esterase‐catalyzed hydrolysis of QM to QTP in controlling the drug release kinetics. That is, the rate‐limiting step in the drug release of F2 and F3 is the conversion rate from QM to QTP. The hydrolyzed QTP has higher water solubility, allowing it to diffuse more readily from the NSPs to the media than the lipophilic QM.

### 2.4 Cell Viability

**Figure 4:**
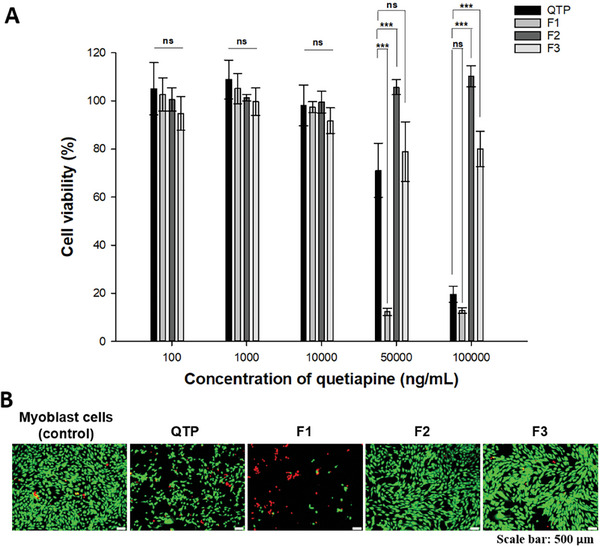
Cytotoxicity of three NSPs (F1–F3). A) Cell viability after treatment with the QTP solution and three NSPs (F1–F3) at different QTP concentrations (100, 1000, 10 000, 50 000, and 100 000 ng mL^−1^) for 24 h. B) Live/dead images of cell viability after treatment with QTP solution and three NSPs at 100 000 ng mL^−1^ for 24 h. Data are expressed as mean ± standard deviation (*n* = 6). Significant differences were analyzed using one‐way analysis of variance (ANOVA), followed by the Bonferroni post hoc test: ***p < 0.001, **p < 0.01, and *p < 0.05; ns: no significant difference (*p* > 0.05).

As the proposed long‐acting formulations were administered by IM injection, a cell viability test was performed with myoblast cells—muscle progenitor cells—to predict the biocompatibility and relative toxicities of the formulations. A significant relationship has been reported between the irritation levels elicited by drug formulations and the extent of myoblast cell damage.^[^ ^32^, ^33^ ^]^ The in vitro viability of myoblast cells was assessed following treatment with the formulations at different concentrations relative to the therapeutic dose of QTP (100 ng mL^−1^).^[^ ^34^ ^]^ No cytotoxicity was observed for the three NSPs (cell viability > 80%) at 10 000 ng mL^−1^, 100 times higher than the therapeutic dose. LAIs require high doses to maintain drug concentrations above the minimum therapeutic concentration over a long period.^[^ ^3^ ^]^ Therefore, higher doses of 50 000 and 100 000 ng mL^−1^, at which QTP previously exhibited a cytotoxic effect, were investigated.^[^ ^35^, ^36^ ^]^ At these concentrations, F1 induced concentration‐dependent toxicity, whereas F2 and F3 were relatively compatible with myoblast cells (**Figure** **4**A). The low cell viability after incubation with high F1 concentrations was likely due to its large initial drug release profile (i.e., ≈80% of QTP released in the first 24 h). Therefore, the high QTP concentrations in the media caused toxicity to myoblast cells. Moreover, the presence of high polymer (PLGA, PVA) concentrations can contribute to the high cytotoxicity of F1. In contrast, F2 and F3, with controlled drug release profiles, did not significantly impact cell viability, even at a high concentration of 100 000 ng mL^−1^. The relatively lower cell viability of F3 (≈70%) compared to F2 (≈90%) can be explained by the pH‐responsive precipitation of QMN in the media. That is, the QM deposited at the bottom of the well could locally interfere with the viability of myoblast cells, while QM‐loaded PLGA nanoparticles were dispersed in the media. Figure 4B shows the fluorescence images of a live/dead assay of C2C12 cells treated with samples at 100 000 ng mL^−1^ after 24 h of incubation. The presence of live cells (green) was proportional to the data shown in Figure 4A. These results demonstrate that the formulations loaded with QM (F2 and F3) can avoid cellular toxicity at high doses.

### 2.5 In Vivo Pharmacokinetic Evaluation in Beagle Dogs

#### 2.5.1 Plasma Concentration–Time Profile

**Figure 5:**
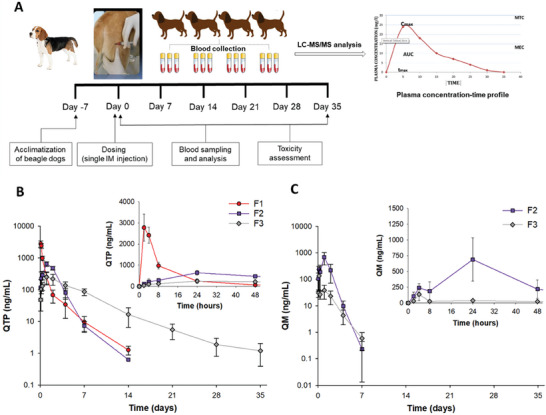
Pharmacokinetic profiles following IM injection of three NSPs (F1–F3) to beagle dogs (35 mg kg^−1^ as QTP). A) Experimental schedule for the in vivo study. B,C) Plasma concentration–time profiles of QTP (B) and QM (C). Data represents mean ± standard deviation (*n* = 4).

**Figure** **5**A illustrates the experimental schedule for the in vivo study. Beagle dogs (*Canis familiaris*) were selected as a model due to their common use in previous studies to investigate the pharmacokinetics of other LAI formulations. In comparison with small animals (mice or rats), beagle dogs exhibit relatively similar metabolic rates with humans that affect the prodrug hydrolysis, drug release, and drug clearance; hence, the pharmacokinetic profiles obtained from the beagle dog model provide valuable information before further clinical evaluation of the new long‐acting formulation.^[^ ^37^, ^38^ ^]^ To maintain the therapeutic effect without toxicity concerns, LAIs should provide plasma QTP concentrations within the therapeutic window (20–300 ng mL^−1^) over an extended period.^[^ ^39^, ^40^ ^]^ PLGA‐based formulations (F1 and F2) released QTP for two weeks after IM injection in beagle dogs; however, the concentrations dropped below the MEC within one week (Figure 5B). A similar pharmacokinetic profile was seen when PLGA microspheres with an average particle size of 8.86 µm loaded with norquetiapine were intramuscularly injected in rats.^[^ ^9^ ^]^ In contrast, F3 established sustained plasma QTP concentrations within the therapeutic window for approximately two weeks and remained above the limit of quantitation (LOQ) for five weeks.

The drug release rate of F2 in vivo was considerably faster than the monthly in vitro controlled drug release profile due to the relatively rapid absorption of 200‐nm PLGA nanoparticles into the systemic circulation. Hydrophilic and small PLGA nanoparticles can diffuse from the site of injection through the interstitial space (≤1 µm) to reach the blood or lymphatic capillaries following IM injection in beagle dogs.^[^ ^5^, ^41^ ^]^ By contrast, QMN underwent pH‐responsive structural rearrangement to form nanoaggregates at physiological pH, retarding the absorption of QM from the injection site into the systemic circulation. For the prodrug in F2 and F3, QM plasma concentration peaked within 24 h postinjection and significantly decreased below the LOQ (0.5 ng mL^−1^) after seven days (Figure 5C). The gradual conversion from QM to QTP by plasma esterase was similar to that of the in vitro dissolution test with added esterase.

#### 2.5.2 Pharmacokinetic Parameter Analyses

The three formulations had similar area‐under‐the‐plasma concentration (AUC_0–35 days_) and extrapolated AUC (AUC_0–∞_) values, representing the equivalent administered dose among the three test formulations (**Table** **2**). The time required to reach the maximum plasma concentrations (*T*_max_) of the three NSPs was relatively short due to the high nanoparticle surface‐area‐to‐volume ratio, ranging from 2 h (F1) to 24 h (F2 and F3) and resulting in rapid drug release. A rapid increase in QTP plasma concentration is necessary to achieve a quick onset of therapeutic effect; however, safety concerns related to the initial burst release must be considered. The dissolution profile of F1 in beagle dogs following IM injection showed an extremely large burst release, with a maximum plasma concentration (*C*_max_) of 2770 ng mL^−1^, nearly 10 times higher than the minimum toxic concentration (MTC) of 300 ng mL^−1^. Formulation F2 reduced the plasma QTP concentration on the first day after injection, with a *C*_max_ of 644.4 ng mL^−1^. However, the high plasma QM concentration at 24 h (≈700 ng mL^−1^) indicated a huge burst effect for PLGA‐QM NSPs, although to a lesser extent than PLGA‐QTP NSPs. The instability of PLGA‐QTP NSPs, leading to particle aggregation at the injection site caused by serum protein adsorption, might account for the unexpectedly longer terminal half‐life (*T*_1/2_) of F1 than F2. In contrast, F3 avoided the huge initial burst of PLGA NSPs with the *C*_max_ of QTP (249.8 ng mL^−1^) and QM (135.8 ng mL^−1^) lower than the MTC. The rapid pH‐responsive structural rearrangement to form nanoaggregates and the relatively high viscosity of QMN could improve drug retention at the injection site and reduce the initial burst release of F3. Additionally, the *T*_1/2_ of QMN was 105.4 h, approximately twofold and fivefold higher than that of PLGA NSPs loaded with QTP (56.7 h) and QM (22.3 h), respectively.

**Table 2:** Pharmacokinetic parameters of QTP in plasma and observed adverse side effects following IM administration of three NSPs (F1–F3) to beagle dogs (35 mg kg^−1^ as QTP).

Formulations		F1	F2	F3
**Pharmacokinetic parameters** ^a^ ^)^	*T*_max_ [h]	2	24	24
	*C*_max_ [h]	2770.0 ± 637.9^***^	644.5 ± 107.2^**^	249.8 ± 99.9
	*T*_1/2_ [h]	56.7 ± 14.2^**^	22.3 ± 5.7^***^	105.4 ± 11.3
	AUC_0–35 days_ [ng h mL^−1^]	34067.6 ± 3868.8^#^	39153.8 ± 4845.8^#^	37255.0 ± 5559.5
	AUC_0–∞_ [ng h mL^−1^]	34163.7 ± 3867.4^#^	39309.4 ± 4915.2^#^	37454.2 ± 5521.2
**Adverse side effects** ^b^ ^)^	Limping	+ (3/4)	+ (1/4)	–
	Vomiting	–	+ (1/4)	–

^a)^ Data are expressed as mean ± standard deviation (*n* = 4). Significant difference analyzed by unpaired two‐tailed Student's *t*‐test compared with F3: ^**^ *p* < 0.01; ^***^ *p* < 0.001; ^#^no significant difference (*p* > 0.05);

^b)^ Adverse side effect was observed (+) with frequency provided in parentheses, or no adverse side effects were observed (–).

Based on the pharmacokinetic profiles, F3 is deemed a potential LAI with an expected similar plasma concentration–time profile following IM administration between beagles and humans due to the relatively similar metabolic rates.^[^ ^37^, ^38^ ^]^ However, the pharmacokinetic parameters, such as *C*_max_, *T*_max_, *T*_1/2_, and AUC, might differ due to differences in body fat distribution, muscle mass, physical activity, and carboxylesterase enzymatic activity between humans and dogs.^[^ ^37^, ^42^ ^]^ Notably, human esterase activity is weaker than that of dogs,^[^ ^8^, ^42^ ^]^ likely resulting in a slower conversion rate from QM to QTP and a slower drug release rate. Therefore, the *T*_max_ and *T*_1/2_ of QMN are expected to be higher in humans than in dogs. These differences might be advantageous for LAI formulations, as the drug concentration is maintained above the MEC over a longer period of time.

#### 2.5.3 Side Effects Assessment

The most commonly observed adverse event was limping on the injected limb following the IM injection of the three NSPs (Table 2). Three out of the four dogs experienced limping lasting for 1–3 days following the administration of F1, which was more severe than that in F2, with the one affected dog recovering after one day. However, one out of three dogs vomited following IM dosing of F2, raising concerns about toxicity despite the low representativeness. Interestingly, F3 was locally tolerable without any observed adverse side effects. The limping side effect likely correlated with the large initial burst release in which the rapid increase in the QTP and QM concentrations exceeded the toxicity level and impaired local tissues. In addition, the high concentrations of polymers (PLGA, PVA) in F1 and F2 might cause limping or vomiting in dogs. Accordingly, the lessened burst release is critical to avoid toxicity, and the simple formulation design of QMN without polymers and surfactants is preferable in terms of the safety profile.

## 3 Conclusion

In this study, we investigated the physicochemical characteristics and in vivo performance of the novel pH‐responsive NSP (QMN) and conventional PLGA NSPs loaded with QTP or QM. The small particle size (150–250 nm) and shear‐thinning rheological property allowed QMN injection using a thin‐gauge needle (26G) with minimal invasiveness and discomfort. QMN offers a simple formulation design and manufacturing process, overcoming many limitations of PLGA systems, with high drug content, high loading efficiency, good physical stability, low toxicity, and minimized initial burst release. The pH‐responsive structural rearrangement of QMN to form nanoaggregates at the injection provides a five‐week sustained release, with two weeks of QTP concentration maintained within the therapeutic window. Taken together, QMN is a potential LAI formulation for less frequent dosing of QTP from once to twice per month. The pH‐responsive NSPs based on the fattigation platform can be applied to develop LAIs of other drugs to improve patient compliance.

## 4 Experimental Section

### Materials

QTP fumarate (USP) was purchased from Aurobindo Pharma, India. PLGA (Resomer RG 502H, acid terminated, molecular weight 7000–17 000) and poly(vinyl alcohol) (PVA; 87–90% hydrolyzed, average molecular weight 30 000–70 000) were purchased from Sigma‐Aldrich (Seoul, Korea). Esterase from porcine liver (lyophilized powder, ≥15 U mg^−1^ solid) was purchased from Sigma‐Aldrich. High‐performance liquid chromatography (HPLC)‐grade solvents, including methanol (MeOH), acetonitrile (ACN), and ethyl acetate (EtAc) were supplied by Honeywell Burdick & Jackson (Korea). PBS tablets, Dulbecco's modified Eagle's medium (DMEM), FBS, and penicillin/streptomycin were purchased from Sigma‐Aldrich (Seoul, Korea). All other chemicals and reagents used in this study were of analytical grade.

### Synthesis of QM

The synthesis of QM has been described in the previous study^[^ ^15^ ^]^ and is summarized in Scheme 1. Briefly, QTP (3.83 g, 10 mmol) and myristic acid (2.51 g, 11 mmol) were dissolved in tetrahydrofuran (THF, 10 mL) before adding 1‐ethyl‐3‐(3‐dimethylaminopropyl) carbodiimide hydrochloride (EDC HCl, 2.88 g, 15 mmol) and 4‐dimethylaminopyridine (DMAP, 0.24 g, 2 mmol). The reaction mixture underwent 24 h reaction at room temperature, followed by column chromatography to yield the purified QM. Table S2 (Supporting Information) provides the physicochemical and biological properties of QTP and QM.

### Preparation of Injectable NSPs

PLGA NSPs loaded with QTP (PLGA‐QTP NSPs, F1) or QM (PLGA‐QM NSPs, F2) were prepared by an emulsification–diffusion method using a high‐speed homogenizer to obtain the desired NSP system.^[^ ^43^ ^]^ Briefly, QTP/QM (equivalent to 100 mg QTP) and PLGA (200 mg) were dissolved in 4 mL of EtAc and added to 8 mL of an aqueous solution containing 2% (w/v) PVA as a stabilizer. The mixture was emulsified using a high‐speed homogenizer (Homogenizer HG‐15D; Daihan Scientific, Seoul, Korea) at 15 000 rpm for 30 min. The resulting pre‐emulsion was added to 12 mL of PVA 2% solution and stirred for 24 h at 20–30 °C in a fume hood to evaporate the organic solvent. The NSPs were then purified by a centrifugation step (15 min, 13 000 rpm, and 25 °C) and resuspended in deionized water (1 mL). The preparation steps were repeated to prepare a larger amount of nanosuspensions, and all samples were combined.

The preparation of QMN (F3) was simple and scalable, using the pH‐triggered self‐assembly method described in our previous study with slight modifications.^[^ ^15^ ^]^ Briefly, QM (equivalent to 100 mg mL^−1^ QTP, 0.26 M) was solubilized and protonated in a mixture of HCl 0.13 M (pH modifier) and THF (volume ratio 1:1). The organic solvent was evaporated for 24 h at room temperature in a hood to obtain the QMN. The final NSPs (F1–F3) were added with 10% (w/v) mannitol as a cryoprotectant and frozen at −80 °C for 24 h and freeze‐dried for 48 h to obtain the powdered injectable dosage form. Prior to injection, the lyophilized powders of the three formulations were reconstituted in sterile deionized water to achieve the aqueous nanosuspensions at the desired concentration (70 mg mL^−1^). The detailed compositions of the three formulations are presented in Table S3 (Supporting Information).

### Particle Size, Zeta Potential, and Morphology of NSPs

The hydrodynamic nanoparticle diameter and polydispersity index (PDI) were determined via dynamic light scattering (DLS) using an ELSZ‐2000 instrument (Otsuka Electronics, Japan). The zeta potential was measured by laser Doppler microelectrophoresis using the same instrument to determine the surface charge of the nanoparticles. Before measurement, the samples were diluted in 2 mL of deionized water. The measurements were repeated thrice, and the average ± standard deviation was determined. The morphology of the nanoparticles was analyzed using FE‐TEM. The NSPs were negatively stained with 2% phosphotungstic acid (w/v) on 200 mesh carbon‐coated copper grids for TEM imaging. The excess sample was removed with filter paper and subsequently dried overnight at room temperature before being processed for imaging using FE‐TEM (Tecnai‐G2 F30 S‐Twin) operating at 300 kV with a 0.20 nm point resolution.

### Physical Stability of NSPs in Storage Conditions

The stability of the three formulations was evaluated in lyophilized solid and aqueous liquid states during storage by measuring the particle size using the DLS method. Freeze‐dried powder samples were stored at 25 °C for one, two, and four weeks and reconstituted in deionized water prior to evaluation. For the liquid state, the average diameter of NSPs was monitored over 96 h at 25 °C. The experiments were performed in triplicate, and the average values were reported.

### Viscosity Measurement and Injectability Test

Shear viscosity was measured with a rotational rheometer (AR‐G2, TA Instruments) equipped with a cone‐and‐plate geometry at 25 °C. The diameter and angle of the cone were ˀ60 mm and 1°, respectively. The gap between the cone and plate was fixed at 32 µm for all measurements. The viscosity at different shear rates (0.1–1000 s^−1^) was determined to create the flow curve for the shear‐dependent viscosity of the NSPs. The measurements were conducted thrice to acquire statistically reliable data, and the shear viscosity was determined as an average value. The injectability test used a 26G, ½‐inch needle (outer diameter: 0.464 mm, inner diameter: 0.260 mm) provided by Korea Vaccine Co., Ltd., Seoul, Korea. A formulation was considered “injectable” if it could easily pass through the needle without exerting considerable effort.^[^ ^6^ ^]^

### Drug Content and Loading Efficiency Determination

The direct method was used to determine drug content and loading efficiency.^[^ ^44^ ^]^ Drug content referred to the percentage of drug weight over the total solid content while loading efficiency was the percentage of QTP successfully formulated into the NSP relative to the initial drug added. The freeze‐dried samples without mannitol were accurately weighed and dissolved in 1 mL of ACN. The mixture was centrifuged at 10 000 rpm for 10 min, and the supernatant was diluted with ACN before HPLC analysis. Drug content and loading efficiency were calculated using the following equations, and all experiments were performed in triplicate:

(1) $$Drug\;content\;\left(\%\right)=\frac{Mass\;of\;QTP\;in\;NPs}{Mass\;of\;NPs\;}\times 100$$

(2) $$Loading\;efficiency\;\left(\%\right)=\frac{Mass\;of\;QTP\;in\;NPs}{Mass\;of\;total\;QTP\;added\;}\times 100$$

### Quantitative Analysis of QTP and QM

An HPLC system (Agilent 1200, Agilent Technologies, USA) with an ultraviolet detector and Empower software was used to measure the drug concentrations. QTP and QM were separated on a Hypersil gold 5 µm C18 column (250  mm × 4.6 mm) and detected at 230 nm using isocratic elution with a flow rate of 1.3 mL min^−1^. The mobile phase used for QTP quantification comprised 54% methanol, 7% ACN and 39% dibasic ammonium phosphate solution (2.6 g L^−1^). The mobile phase for QM was a mixture of ACN and 0.1% trichloroacetic acid (70:30). The drug content was determined relative to the peak areas of the drug standards (0.001–0.05 mg mL^−1^) in MeOH.

### pH‐Responsive Property of QMN

The pH of each sample was determined using a calibrated pH meter (A211, Thermo Fisher Scientific). This investigation was repeated thrice, and the average ± standard deviation was determined. The effect of pH on the physical appearance of the three NSPs was investigated by injecting 0.1 mL of NSPs into 0.5 mL of pH 4.5 and 7.4 buffers. After 24 h of stabilizing, the physical state of NSPs, either dispersed or aggregated, was observed. The particle size and morphology of the nanoparticles in different pH buffers were analyzed using FE‐SEM. The NSPs were placed on 200 mesh carbon‐coated copper grids and washed with an excess volume of the pH buffers. The grids were then mounted on carbon tape and sputter‐coated with a gold/palladium alloy before imaging using FE‐SEM (JEOL, JSM‐7900F, Japan) at 5 kV and room temperature.

### NSP Stability in Salt and Serum Conditions

The stability of the three nanosuspensions in different salt concentrations (NaCl 0.09, 0.9 and 9%, w/v) and serum condition (FBS 10% v/v in PBS 0.01 M) were evaluated. To assess salt stability, the three formulations (0.1 mL) were added to 1 mL of NaCl solutions and stabilized at 37 °C for 24 h before analysis. Similarly, the formulations (0.5 mL) were mixed with 5 mL of FBS 10% (in PBS 0.01M) and incubated at 37 °C over 96 h. The particle size of samples was measured at predetermined times using the DLS method.

### In Vitro Drug Release Studies of NSPs

The in vitro drug release profile of NSPs (F1–F3) was evaluated using a dialysis method.^[^ ^1^, ^45^ ^]^ Dissolution tests were performed in the absence and presence of esterase in the dissolution media to evaluate the effect of enzyme‐oriented hydrolysis on the dissolution profile of the lipophilic compound.^[^ ^30^ ^]^ To guarantee the sink condition of the dissolution medium, 0.5% (w/v) polysorbate 80 was added to 0.01 M PBS (pH 7.4), and 0.1% sodium azide was added as an antimicrobial agent. For the dissolution test in the presence of esterase, 5 U mL^−1^ esterase was added to the medium. NSPs containing QTP or QM (equivalent to QTP 5 mg) in 1 mL of media were placed in a 3 mL dialysis tube (Pur‐A‐Lyzer, molecular weight cut‐off (MWCO) 3500 Da), which was placed in a 50 mL tube containing 40 mL of the release medium. The tubes were incubated at 37 °C in a shaking bath at 100 rpm. At predetermined time points, 1 mL of the medium was withdrawn, and 1 mL of fresh buffer was added. The samples were diluted with the mobile phase, and the concentrations of QTP and QM in the aliquot were determined by HPLC.

### Cell Viability

Cell viability was assessed using WST‐1 and live/death assays to investigate the cellular toxicity of the NSPs. The mouse myoblast cell line (C2C12) was obtained from the Korean Cell Line Bank (Seoul, Korea), which was cultured in DMEM (high glucose) supplemented with 10% FBS and 1% penicillin/streptomycin and maintained in a humidified incubator at 37 °C with 5% CO_2_. The harvested cells were seeded in a 96‐well plate at a density of 10^4^ cells per well. After 24 h of incubation, the medium was replaced with fresh DMEM containing QTP and NSPs (QTP: 100–100 000 ng mL^−1^) at 37 °C. After incubating for 24 h, the medium was replaced with fresh media containing 50 µL of WST‐1 agent (EZ‐Cytox, Dogen, Korea) and incubated for 2 h at 37 °C. The absorbance of the cell culture media was measured at 450 nm using a microplate reader (Synergy H1, Biotek, USA). The control samples were incubated in DMEM without treatment. Cell viability was calculated using the following equation:

(3) $$\mathrm{Cell}\;\mathrm{viability}\;\left(\%\right)=\frac{At-Ab}{\mathrm{Ac}-\mathrm{Ab}}\times 100$$

where Ab denotes absorbance of blank (culture medium without cell), At denotes absorbance of test samples (culture medium and test solution), and Ac denotes absorbance of positive control (culture medium without test solution).

To investigate the live/dead cell proportion, culture media was removed and replaced with live/dead working solution prepared using the LIVE/DEAD Viability/Cytotoxicity assay kit (Invitrogen, Thermo Fisher Scientific, USA) according to the manufacturer's protocol. The images were captured using a fluorescence microscope (Leica Microsystems, Germany).

### In Vivo Pharmacokinetic Evaluation in Beagle Dogs

The in vivo performance of the three formulations was evaluated following IM injection to normal beagle dogs (*C. familiaris*) after approval by the Animal Experimentation Ethics Committee of Ndic Co., Ltd., based on the Animal Protection Act (Approval number: P 233032). Male beagle dogs (2–3 years old, 10 ± 1 kg) were acquired from Ndic Co., Ltd. (Gyeonggi‐do, Korea) and housed in a temperature‐ and relative humidity‐controlled room (21 ± 2 °C and 50 ± 15%, respectively) with a 12 h light‐dark cycle. The dogs were acclimatized to the environment for one week before testing. During the test period, ≈250 g of solid feed was fed into the feed box every afternoon. The dogs were then randomly divided into three groups (*n* = 4 per group), and the formulations were injected into a single left hind leg of each dog at a QTP concentration of 35 mg kg^−1^ using a 26G needle. To determine the drug concentration in the plasma, blood samples (≈3 mL) were collected from the jugular vein using a 26G heparinized syringe at predetermined times (0 h (predose), 2, 4, 8, 24, 48, 96, 168, 336, 504, 672, and 840 h postinjection). The collected blood was immediately centrifuged at 4000 rpm for 10 min at 4 °C. The separated supernatant was stored at −70 °C until quantitative analysis. Plasma proteins were precipitated by mixing 20 µL of samples with 180 µL of ACN containing the internal standard (carbamazepine 1 ng mL^−1^); the mixture was centrifuged to collect the supernatant (150 µL) for quantitative analysis. Plasma QTP and QM concentrations were determined using a validated HPLC‐MS/MS assay.

The 35 mg kg^−1^ QTP dose administered to the dogs was estimated based on an oral QTP dose of 500 mg day^−1^, assuming 100% bioavailability for the parenteral route.^[^ ^46^ ^]^ The correction factor (Km) was used to calculate the human equivalent dose from the animal dose and vice versa, based on the weight and body surface area:^[^ ^47^ ^]^

(4) $$\begin{array}{ccc} & & \mathrm{Monthly}\;\mathrm{animal}\;\mathrm{dose}\\ & & \;=\frac{\mathrm{oral}\;\mathrm{dose}\;\left(\frac{\mathrm{mg}}{\mathrm{day}}\right)\times \mathrm{oral}\;\mathrm{bioavailability}\left(\%\right)\times 28\;\left(\mathrm{days}\right)}{\mathrm{human}\;\mathrm{body}\;\mathrm{weight}\;\left(\mathrm{kg}\right)}\\ & & \;\times \;\mathrm{Km}\;\left(1.8\;\mathrm{for}\;\mathrm{dogs}\right)=\frac{500\times 9\%\times 28}{60}\times 1.8\approx 35\;\left(\mathrm{mg}/\mathrm{kg}\right) \end{array}$$

Pharmacokinetic parameters such as AUC_0–35 days_, AUC_0–∞_, *C*_max_, *T*_max_, and *T*_1/2_ were calculated using the WinNonlin program (Pharsight Co., Inc., USA). All experimental results obtained in the experiment are expressed as mean values ± standard deviation, and the noncompartment model was applied as the drug compartment model. Clinical symptoms related to adverse side effects, such as changes in movement, were investigated from the start of drug administration to the 35^th^ day of administration.

### Statistical Analysis

All details regarding sample size, data presentation, statistical analysis, and significant differences are provided in the relevant figure captions. Statistical analysis was performed using SigmaPlot (version 14.0; Systat Software, San Jose, CA, US). Unpaired two‐tailed student's *t*‐test was used for two‐group comparisons. One‐way analysis of variance (ANOVA), followed by Bonferroni post hoc analysis, was used for multigroup comparison. Differences between samples were considered significant at a *p*‐value < 0.05. The data are presented as mean ± standard deviation.

## Conflict of Interest

The authors declare no conflict of interest.

## Supporting information

Supporting Information

Supporting Information

Supporting Information

Supporting Information

## Data Availability

The data that support the findings of this study are available from the corresponding author upon reasonable request.
