# Supplementary material for: Novel pH‐Responsive Structural Rearrangement of Myristic Acid‐Conjugated Quetiapine Nanosuspension for Enhanced Long‐Acting Delivery Performance
Source: Adv Sci (Weinh). 2024 Sep 3;11(40):2405200. doi: 10.1002/advs.202405200 (PMC11516153; doi:10.1002/advs.202405200)
Supplement: Supplementary file 1 — Supporting Information [file ADVS-11-2405200-s001.docx]

**Supporting Information**

**Novel pH-Responsive Structural Rearrangement of Myristic Acid-Conjugated Quetiapine Nanosuspension for Enhanced Long-Acting Delivery Performance**

*Hy Dinh Nguyen, Hai Van Ngo, Beom-Jin Lee^*^*

H. D. Nguyen, H. V. Ngo, B. -J. Lee

College of Pharmacy, Ajou University, Suwon 443-749, Republic of *Korea*

E-mail: [bjl@ajou.ac.kr](mailto:bjl@ajou.ac.kr)

**Table S1.** Drug content and loading efficiency at different initial ratios (w/w) between quetiapine (QTP)/ quetiapine myristate (QM) and poly(lactide-co-glycolide) (PLGA)**.**

| **Initial amount of drug [mg]  added to 200 mg PLGA^a)^** | | **Drug content [%]^b)^** | **Loading efficiency [%]^b)^** |
| --- | --- | --- | --- |
| QTP | 60 | 13.03 ± 0.53 | 57.23 ± 1.00 |
|  | 80 | 16.39 ± 0.38 | 61.47 ± 1.44 |
|  | 100 | 20.12 ± 0.89 | 67.18 ± 1.14 |
| QM | 92.8 (equivalent to 60 mg QTP) | 21.89 ± 0.21 | 93.42 ± 0.26 |
|  | 123.8 (equivalent to 80 mg QTP) | 23.45 ± 1.49 | 95.50 ± 0.09 |
|  | 154.8 (equivalent to 100 mg QTP) | 27.92 ± 1.44 | 95.36 ± 0.04 |

^a)^ Different initial amounts of QTP or QM (equivalent to 60, 80, or 100 mg QTP) were added to 200 mg of PLGA to evaluate the drug loading and loading efficiency of QTP and QM into PLGA NPs. ^b)^ Data are expressed as mean ± standard deviation (*n* = 3).

**Table S2.** Physicochemical and biological properties of QTP fumarate and QM**.**

| **Factor** | **QTP** | **QM** |
| --- | --- | --- |
| Physical appearance | White solid (fumarate salt) | Yellowish viscous oil |
| Molecular weight [g mol^-1^] | 383.5 | 593.4 |
| Chemical formula | C_21_H_25_N_3_O_2_S | C_35_H_51_N_3_O_3_S |
| Solubility in water | 49.4 mg L^-1^ | 0.45 mg L^-1^ |
| pKa | 3.3, 6.8 | - |
| log P | 2.81 | 6.13 |
| Drug class | Atypical antipsychotics | Prodrug of quetiapine |
| Route of administration | Oral tablet  [150-800 mg day^-1^] | Long-acting injectable (IM) |
| Half-life | 6-7 h | - |
| Excretion | Liver | - |
| Oral bioavailability | 9% | - |

**Table S3.** Composition of three aqueous nanosuspensions (F1-F3).

| **Formulation** | **F1 (PLGA-QTP)** | **F2 (PLGA-QM)** | **F3 (QMN)** |
| --- | --- | --- | --- |
| Lyophilized powder [mg] | 450 | 350 | 210 |
| Sterile water [mL] | 1 | 1 | 1 |
| Concentration of QTP/QM [mg mL^-1^] | 70 | 108.5^a)^ | 108.5^a)^ |

^a^^)^ QM concentrations in F2 and F3 were equivalent to 70 mg mL^-1^ QTP.


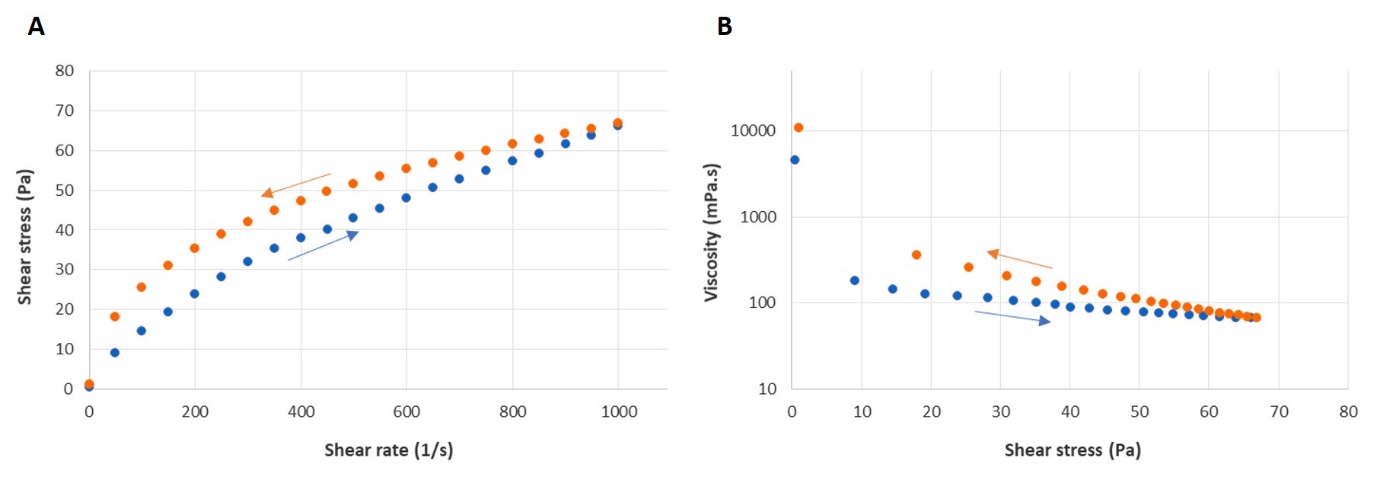


Figure S1. Flow curve of shear stress versus shear rate (A) and viscosity versus shear stress for QMN (F3) at 25 ^o^C. The upward and downward arrows refer to increasing and decreasing shear rate, respectively.

**Video S1.** Injectability test of three nanosuspensions (F1-F3) using 26 G needle. All three nanosuspensions could easily pass through the needle without exerting much effort (injectable) using the 26 G needle.
